# Schottky Barrier Height and Image Force Lowering in Monolayer MoS_2_ Field Effect Transistors

**DOI:** 10.3390/nano10122346

**Published:** 2020-11-26

**Authors:** Yonatan Vaknin, Ronen Dagan, Yossi Rosenwaks

**Affiliations:** School of Electrical Engineering, Tel-Aviv University, Tel Aviv 69978, Israel; yhonatan.v@gmail.com (Y.V.); ronendagan@gmail.com (R.D.)

**Keywords:** Schottky barrier height, image-force, TMD, KPFM, 2D materials, FET

## Abstract

Understanding the nature of the barrier height in a two-dimensional semiconductor/metal interface is an important step for embedding layered materials in future electronic devices. We present direct measurement of the Schottky barrier height and its lowering in the transition metal dichalcogenide (TMD)/metal interface of a field effect transistor. It is found that the barrier height at the gold/ single-layer molybdenum disulfide (MoS_2_) interfaces decreases with increasing drain voltage, and this lowering reaches 0.5–1 V We also show that increase of the gate voltage induces additional barrier lowering.

## 1. Introduction

Since the introduction of the first single-layer molybdenum disulfide (MoS_2_) field effect transistor (FET) by Andras Kis et al. [1], in 2011, the transition metal dichalcogenides (TMDs) were proved to be beneficial as single-layer semiconductors for the post-silicon era due to their mechanical flexibility [2], high mobility and on:off ratio [3,4,5], immunity to short channel effects [6], band gap suitability to the visible-near IR spectrum [7], and abrupt switching capabilities [8]. MoS_2_, in particular, is considered applicable for a vast range of microelectronics [5,9], optoelectronics [10,11], sensing [12,13], spintronics [14], solar cells [15] and flexible electronics [16,17].

One of the main parameters governing the transistor characteristics, and correspondingly its potential applications, is the nature of the metal—semiconductor contact [18]. A Schottky barrier will be formed at such interfaces in the presence of a higher metal work function, as introduced by the Schottky–Mott theory [19,20]. Interface states also play a crucial role in Schottky barrier formation, especially in 2D TMD-based devices, due to the lack of dangling bonds governing the band alignment at the TMD/metal contact [21,22,23]. Many efforts are invested in reducing the Schottky barrier height or even obtaining ohmic contact of such interfaces, by either introducing a doped two-dimensional (2D) TMD layer as an intermediate layer [24] or a mixed transition layer containing van der Waals junctions [23], by using Cobalt as a tunneling barrier between the h-BN Gold electrodes [25], or by implementing Nickel electrodes [26]. Evaluation of the Schottky barrier height (SBH) in such devices can be achieved by either fitting the subthreshold regime transfer characteristics at different temperatures with the thermionic emission law [27,28], introducing radiation and measuring the resulting photocurrent [29,30], conductive atomic force microscopy (C-AFM) measurements [31], or by Kelvin probe force microscopy (KPFM) measurements [23,32]. Both the transfer characteristics fitting, the photoelectric measurement, and the KPFM methods enables device level characterization, including monitoring the terminals dependency of the barrier height.

In this work, we are using the KPFM, equipped with <25 nm radius tip, as it provides direct measurement of the band profile across the device, in contrast to the alternative methods described above; the later provides solely the SBH and not the complete band profile, and are indirect. Moreover, the KPFM feedback nullify the electrostatic force, therefore minimizes tip-induced band bending effects.

Image force barrier lowering is a dominant mechanism in the case of transistor with Schottky contacts, governed by both the drain and source bias, and the gate voltage. In the presence of this mechanism, the barrier height, governing the threshold voltage, and consequently its characteristics, will be modulated [3,29,33]. Schottky diode performance is also effected by the barrier height, as it dictates the reverse saturation current, governed by both diffusion, thermionic emission and tunneling, and hence cause ideality factor (*η*) variations [33]. Moreover, in the case of two-dimensional heterostructures, such as TMD/graphene, the barrier height lowering can be attributed to both graphene quantum capacitance [34,35] and the image force barrier lowering effect [36,37], effecting the on:off ratio of such FET devices. As the number of MoS_2_ layers decreases, the on:off ratio reduction, due to the image force, becomes more significant, as already reported [38,39]. Although the SBH in 2D TMD/metal interfaces has been widely discussed in previous reports [40,41,42,43,44], to the best of our knowledge no direct measurement of the image force barrier lowering effect in thin films has been reported. We present here a direct measurement of the Schottky barrier height and its lowering due to the image force effect, induced by both the gate voltage and the source to drain bias, in single-layer MoS_2_ FETs.

## 2. Materials and Methods

Monolayer MoS_2_ sample was exfoliated from an MoS_2_ crystal supplied by Structure Probe, Inc. (SPI) Supplies (West Chester, PA, USA) using the scotch tape technique [45], and then transferred on top of a 90 nm silicon oxide (SiO*_2_*) die. Alignment marks were patterned on the die prior exfoliation using optical lithography, and optical microscope was used to identify thin MoS_2_ flakes by their contrast. Contact made of 50 nm gold over 3 nm titanium were then designed by E-beam lithography and evaporated using an electron-beam evaporator. Lift-off was then performed using *N*-Methyl-2-pyrrolidone (NMP) at 80 °C. The device was then taped onto a chip carrier using carbon tape, wire bonded and carried into an N_2_ glove box for further annealing and measurements. Electrical measurements, including device characteristics, performed inside the N_2_ glove box, were conducted using a semiconductor parameter analyzer (B1500A, Agilent Technologies, Santa Clara, CA, USA), in addition to KPFM enabling electrostatic potential measurements in-operando. Raman spectra was collected, using a 532 nm laser of the HORIBA LabRAM HR Evolution Raman spectrometer (Kyoto, Japan). A 100× objective was used to obtain <1 μm < spot diameter with 1800 g mm^−1^ grating.

## 3. Results and Discussion

**Figure 1:**
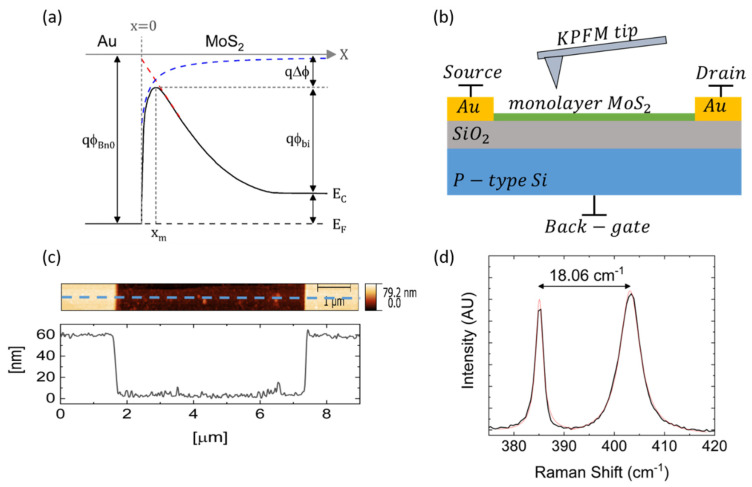
(**a**) Schematic layout of the energy band diagram of a metal/semiconductor junction. The red and blue dashed lines represent the image charges, and the Schottky barrier height contribution to the potential energy of an electron, respectively. (**b**) Schematics of a field- effect transistor (FET) composed of a monolayer molybdenum disulfide (MoS_2_) layer as the channel, drain and source gold terminals, and a back-gate, in addition to the Kelvin probe force microscopy (KPFM) tip. (**c**) An atomic force microscopy (AFM) image, in addition to a topography profile along the dashed blue line, of the single layer MoS_2_ device. (**d**) Raman spectrum, in addition to the fitted peaks, of the single layer MoS_2_ flake, presenting separation of 18.06 (cm^−1^) between $E_{2g}^{1}$ and $A_{1g}$.

When an external field *ψ* is applied to a metal/semiconductor system in the −*x* direction (see Figure 1), the total potential energy of an electron within the semiconductor is given by:ase

(1) $$E\left(x\right)=-\frac{q^{2}}{16\pi \epsilon_{0}\epsilon_{eff}x}-q\left|\psi \right|x$$

where *q* is the electron charge, *ϵ*_0_ is the semiconductor permittivity, *ψ* is the electric field within the semiconductor, and *x* is the distance into the semiconductor away from the interface. *ϵ_eff_* in the above equation represents the effective permittivity of a monolayer semiconductor, calculated as the average of the dielectric constant of its surrounding materials. When an electron is in the vicinity of a metal electrode, an attractive force, known as the image force, will be formed due to the induced positive image charge in the metal. The first term in Equation (1) represents the work required for an electron to overcome this image force, and the second term is the work due to the electric field at the junction, located at *x* = 0, as described by the Schottky—Mott theory [19,20].

The peak position and the barrier lowering are extracted from Equation (1), and given by [18]:

(2) $$x_{m}=\sqrt{\frac{q}{16\pi \epsilon_{0}\epsilon_{eff}\left|\psi \right|}}\;\;\;\;\;\;\;,\;\;\;\;\;\;\;\Delta \phi =\sqrt{\frac{q\left|\psi \right|}{4\pi \epsilon_{0}\epsilon_{eff}}}=2\left|\psi \right|x_{m}$$

where *x_m_* is the peak position, and Δ*ϕ* is the barrier lowering. The difference between the work function of the metal and the semiconductor affinity, denoted as *qϕ_Bn0_* at the metal side in Figure 1a, is composed of: (1) The energy difference between the conduction band and the Fermi level (FL) of the semiconductor; (2) the semiconductor band bending denoted as *qϕ_bi_*; and (3) the image force induced barrier lowering denoted as *q*Δ*ϕ*. The red and blue dashed lines in Figure 1a represent the image charges and the barrier height-induced electric field contribution to the potential energy of the electron as a function of its distance from the metal/semiconductor interface, respectively.

Figure 1b depicts our experimental set up, which is a monolayer MoS_2_ FET composed of a channel, drain and source gold electrodes, and a back-gate, in addition to the measuring KPFM tip. The device was scanned by the KPFM tip, using the dual frequency technique, to simultaneously obtain the topography and the electrostatic potential distribution along the device. An AFM image of the measured FET device, in addition to a topography cross-section is presented in Figure 1c. Figure 1d presents measured (purple) and fitted (pink) Raman spectrum, and shows a separation of 18.06 cm^−1^ between $E_{2g}^{1}$ and $A_{1g}$ corresponding to single layer MoS_2_ [46]. *I-V* characteristics, including *I_d_*(*V_d_*) and *I_d_*(*V_g_*) curves, are presented in Figure S1 of the supporting information.

It should be emphasized that, by definition, the KPFM measurement follows the local vacuum level (LVL). This implies that in the absence of image force barrier lowering, both the LVL and the KPFM measurement represent the difference between the work functions of the metal and the semiconductor. By applying a voltage on one of the metal electrodes (source or drain), the electric field at the interface, and consequently the barrier height, will change; this will be measured by the KPFM as described in details below.

**Figure 2:**
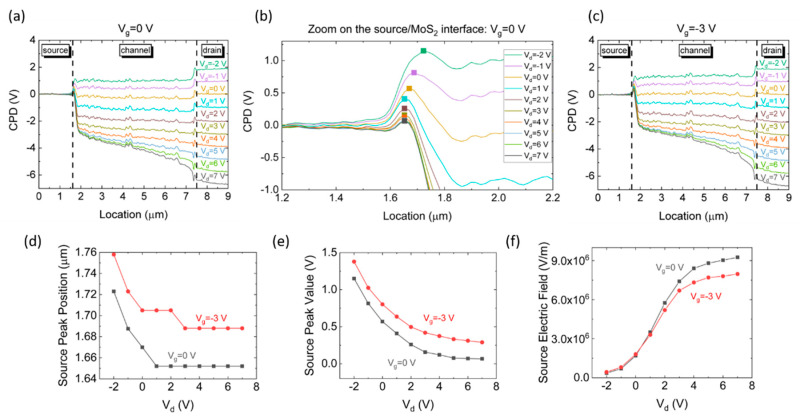
(**a**) Contact potential difference (CPD) measurements of a monolayer molybdenum disulfide (MoS_2_) field-effect transistor (FET), at *V_g_* = 0 V and varying drain voltages *V_d_* = −2 V to *V_d_* = +7 V. (**b**) Closer view of the CPD distribution at the gold/MoS_2_ interface (at the source side). The squares represent the peak point in each curve. (**c**) CPD measurements of the same device, at *V_g_* = −3 V and varying drain voltages *V_d_* = −2 V to *V_d_* = +7 V. (**d**–**f**) Peak position, CPD value at the peak position, and electric field at the gold/MoS_2_ interface, as a function of the applied drain voltage, for both applied gate voltages, respectively.

Figure 2a presents a series of KPFM measurements, showing the contact potential difference (CPD) along the monolayer MoS_2_ FET, for various drain voltages and *V_S_* = *V_g_* = 0 V; a closer view of the CPD profile at the source/MoS_2_ interface is presented in Figure 2b. A depletion region of ~0.2 μm is observed at zero drain bias, in agreement with previous theoretical reports [47,48]. Figure 2c presents a series of KPFM measurements, showing the CPD distribution along the same device for various drain voltages, where *V_S_* = 0 V, and *V_g_* = −3 V. The actual interface position was measured by KPFM to be 1.6 × 10^−6^ m as presented in Figure 1c. The relative peak position was extracted from this measurement and is attached in Figure 2d. The CPD peak potential, relative to the grounded source, is presented in Figure 2e.

The electric field distribution along the device, for both *V_g_* = 0 V and *V_g_* = −3 V, were calculated as the first derivative of the CPD profile measured by the KPFM and are presented in Figure S2 in the supporting information. The electric field at the metal/MoS_2_ interface, as a function of the applied drain voltage, for both gate voltages, is presented in Figure 2f. As the drain voltage increases, the CPD difference at the source/MoS_2_ contact (CPD_source_ − CPD_MoS2_) increases, and consequently the CPD slope from *x_m_* to the conducting channel increases. This CPD change is equivalent to modifying the slope of the dashed red line in Figure 1a. As the drain voltage increases, the electrostatic potential within the semiconductor approaches the drain potential. Since the source electrode is grounded, this potential shift within the semiconductor is added to the initial band bending at the source/MoS_2_ interface. As a result, the electric field at the interface increases, and the corresponding slope of the dashed red line becomes steeper. Therefore, as the image force remains constant, the barrier height becomes lower, and the peak moves towards the source interface. The peak height decrease with increasing drain voltage, is attributed to the segmentation change of the barrier height, *qϕ_Bn_*_0_ *=* *q*Δ*ϕ + qϕ_bi_ + (V_C_ − E_F_)*, at the semiconductor region, as presented above in Figure 1a. The actual band bending of the conduction band *qϕ_bi_* decreases, while the image force induced barrier lowering (*q*Δ*ϕ)* increases.

In order to compare the calculated barrier lowering to the measured one, we assume the following: A gold work function of 5.3 eV [49], MoS_2_ electron affinity of 4 eV [50] and an unintentionally n-type doping of the MoS_2_ [51], inducing an energy difference of 0.2 eV between the conduction band minimum and the FL of the MoS_2_ layer [52]. Consequently, the work function difference, causing the band bending at the gold/MoS_2_ interface, denoted as *q*Δ*ϕ + qϕ_bi_* in Figure 1a, is 1.1 eV. The actual built-in potential, *qϕ_bi_*, is directly extracted from Figure 2a as the potential difference between the MoS_2_ layer and the peak height, and is ~0.7 eV. This means that at *V_Drain_* = 0 V, an initial barrier lowering of 0.4 eV at the gold/MoS_2_ interface will be added to the change in *q*Δ*ϕ* due to the applied source-drain voltage. The FL position, at *V_s_* = *V_d_* = 0 V, is shifted downward from *E_C_* as the gate voltage decreases, due to electrons attraction towards the semiconductor layer. A shift of 0.3 V, between the CPD of the MoS_2_ layer at *V_g_* = 0 V and the CPD level at *V_g_* = −3 V is presented in Figure 2a,c, in agreement with this FL shift. A dedicated scheme, presenting the CPD distribution at *V_d_* = 0 V and in various gate voltages, and highlighting this CPS difference, is presented in Figure S3 of the supporting information. Hence, the initial barrier lowering at *V_Drain_* = 0 V and *V_g_* = −3 V, is higher than at *V_g_* = 0 V, and was estimated to be 0.6 eV.

**Figure 3:**
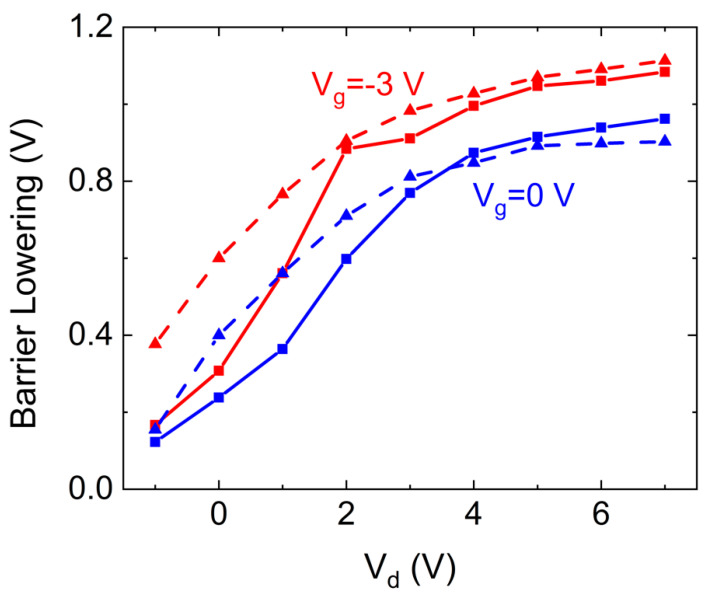
Barrier lowering as function of the applied drain voltage, for three different gate voltages. The squares represent the barrier lowering calculated using Equation (2), and the triangles represent the measured one.

Figure 3 presents a comparison of the barrier lowering, extracted directly from the measured CPD (dashed), and the calculated one using Equation (2) above (solid lines). The comparison is shown for two gate bias, −3 V and 0 V.

By increasing the drain voltage, the electric field increases, the slope of the dashed red line in Figure 1a steepen, the energy at the intersection point decreases, and consequently *q*Δ*ϕ* increases. Further increase of the electric field will result in negligible change of the barrier lowering and the peak position; this is due to the asymptotic behavior of the energy induced by the image charges force presented in Figure 1a. However, the FL position within the band gap and, consequently, the charge concentration within the depletion region are governed by the gate voltage. As the gate voltage increases, electrons are injected into the MoS_2_, and hence less positive charges will be introduced at the depletion region near the gold/MoS_2_ interface. The potential energy associated with the corresponding induced image charges (noted as the first term of Equation (1)) will then decrease. Therefore, the energy at the intersection point increases, and the barrier lowering decreases, as presented. This gate-dependent Schottky barrier height behavior was previously reported by Chen et al. [40]. The barrier height reduction of ~0.7 V at *V_d_* = 1 V and *V_g_* = −3 V, resulting with barrier height of ~0.6 V, is in accordance with previous reports [53,54], presenting mean barrier height of ~0.5 V in top contacted MoS_2_ devices.

Given an ideal Schottky diode, the reverse bias saturation current depends exponentially on the barrier height. By applying positive bias to the diode, the theoretical band bending, denoted as *qΔϕ* + *qϕ_bi_* in Figure 1a, will decrease. As a result, and based on the above observation presented in Figure 3, the barrier lowering induced by the image charges will decrease by approximately 0.1 V. The reverse saturation current will decrease due to the larger barrier height, and the ideality factor will be modulated accordingly. Furthermore, an increase of the gate voltage will reduce the barrier lowering effect as presented, and hence the barrier height will increase. The dark current will then decrease, resulting with increased ideality factor, as presented by Moon et al. [53].

In addition, as the thermionic emission in metal/semiconductor interfaces is exponentially proportional to the barrier height, the resulting on:off ratio in such FET devices depends on the change of the barrier height in those two states. Since the barrier lowering is more dominant at negative gate voltages, as presented in Figure 3, an on:off ratio reduction will be obtained, in agreement with previous reports [37,39,40]. Extraction of the image force barrier lowering contribution to the overall on:off ratio reduction enables further evaluation of the other involved mechanisms in 2D TMD-based FET devices. Moreover, by knowing the energy separation between the FL and the conduction band minimum of a semiconductor, this technique may be used to evaluate the exact Schottky barrier height formed in a certain device.

## 4. Conclusions

In summary, we have used KPFM to measure both the image force barrier lowering effect in addition to the built-in potential in thin-film MoS_2_ FET devices. We have also shown good agreement between the measured change in the barrier modulation and the expected lowering according to the literature. It was found that the Schottky barrier height decreases with increasing gate voltage, consistent with previous reports. Better understanding of the image force barrier lowering effect, as well as evaluation of the Schottky barrier height, in thin-film TMD-based transistors, helps with improving the contact quality of such devices.

## Supplementary Materials

The following are available online at https://www.mdpi.com/2079-4991/10/12/2346/s1. Figure S1. (**a**) *I_d_*(*V_d_*) characteristics. (**b**) *I_d_*(*V_g_*) characteristics. Figure S2. Electric field distribution at the gold/MoS_2_ interface periphery of the source contact, for both *V_Back–gate_* = 0 V and *V_Back–gate_* = −3 V, respectively. The electric fields were calculated as the first derivation of the CPD distribution presented in Figure 2a,c of the manuscript, measured by the KPFM. Figure S3. CPD profile of both *V_g_* = 0 V and *V_g_* = −3 V at *V_d_* = *V_s_* = 0 V presenting separations of 0.3 eV are presented.
