# Supplementary material for: Schottky Barrier Height and Image Force Lowering in Monolayer MoS2 Field Effect Transistors
Source: Nanomaterials (Basel). 2020 Nov 26;10(12):2346. doi: 10.3390/nano10122346 (PMC7761329; doi:10.3390/nano10122346)
Supplement: Supplementary file 1 [file nanomaterials-10-02346-s001.pdf]

nanomaterials

MDPI

Supplementary Material

Schottky Barrier Height and Image Force Lowering in Monolayer MoS_2 Field Effect Transistors

Yonatan Vaknin, Ronen Dagan and Yossi Rosenwaks *

School of Electrical Engineering, Tel-Aviv University, Tel Aviv 69978, Israel; yhonatan.v@gmail.com (Y.V.); ronendagan@gmail.com (R.D.)

* Correspondence: yossir@tauex.tau.ac.il

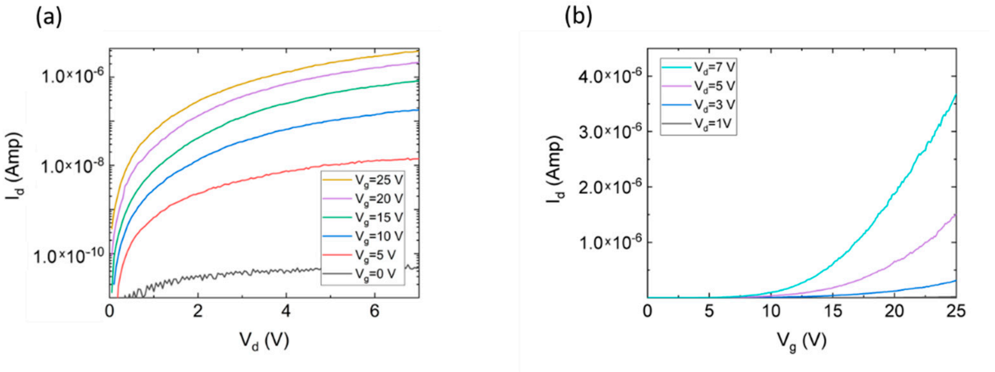

Figure S1. (**a**) $I_d(V_d)$ characteristics. (**b**) $I_d(V_g)$ characteristics.

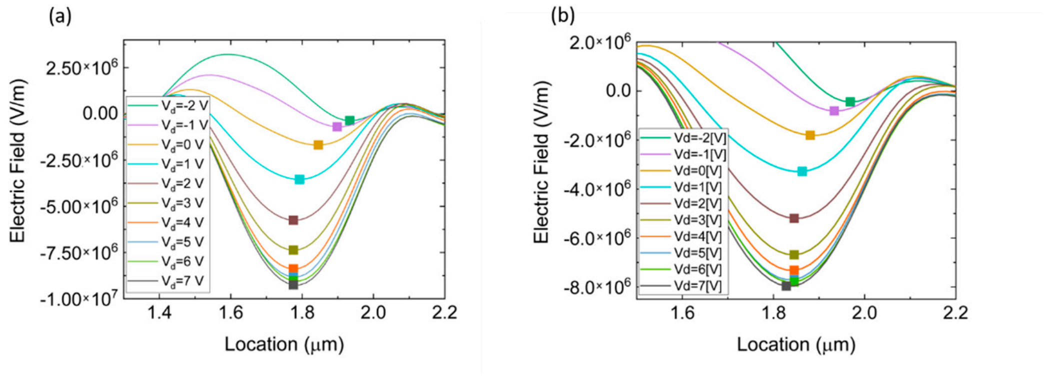

Figure S2. (**a**) and (**b**) Electric field distribution at the gold/MoS_2 interface periphery of the source contact, for both $V_{Back-gate}$ = 0 V and $V_{Back-gate}$ = −3 V, respectively. The electric fields were calculated as the first derivation of the CPD distribution presented in Figure 2a and 2c of the manuscript, measured by the KPFM.

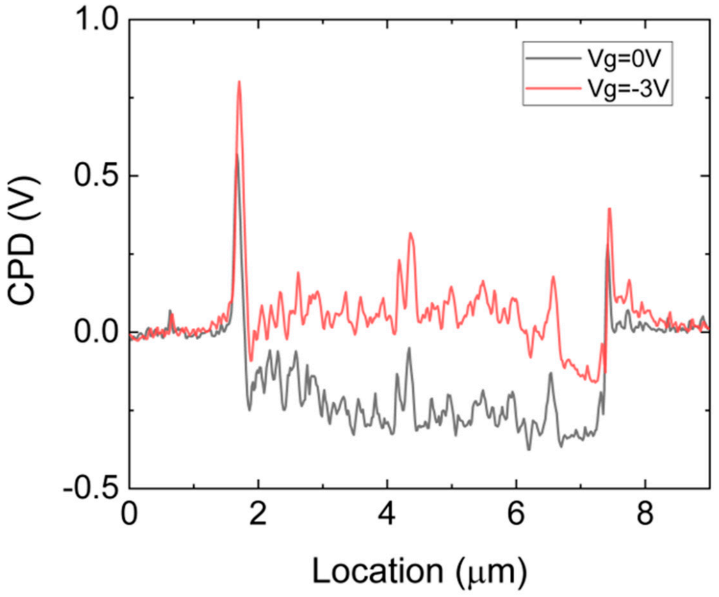

Figure S3. CPD profile of both $V_g = 0$ V and $V_g = -3$ V at $V_d = V_s = 0$ V presenting separations of 0.3 eV are presented.

Publisher's Note: MDPI stays neutral with regard to jurisdictional claims in published maps and institutional affiliations.

© 2020 by the authors. Licensee MDPI, Basel, Switzerland. This article is an open access article distributed under the terms and conditions of the Creative Commons Attribution (CC BY) license (http://creativecommons.org/licenses/by/4.0/).
